# The four Zn fingers of MBNL1 provide a flexible platform for recognition of its RNA binding elements

**DOI:** 10.1186/1471-2199-12-20

**Published:** 2011-05-06

**Authors:** Danielle Cass, Rachel Hotchko, Paul Barber, Kimberly Jones, Devika P Gates, J Andrew Berglund

**Affiliations:** 1 Department of Chemistry, Reed College, Portland, OR 97202, USA; 2 Department of Chemistry and Biochemistry, St. Mary's College of Maryland, St. Mary's City, MD 20686, USA; 3 Department of Chemistry and Institute of Molecular Biology, University of Oregon, Eugene, OR 97403, USA

## Abstract

**Background:**

Muscleblind-like 1 (MBNL1) is an alternative splicing factor containing four CCCH Zinc fingers (ZnFs). The sequestration of MBNL1 by expanded CUG and CCUG repeats is a major component in causing myotonic dystrophy. In addition to binding the structured expanded CUG and CCUG repeats; previous results suggested that MBNL1 binds single-stranded RNAs containing GC dinucleotides.

**Results:**

We performed a systematic analysis of MBNL1 binding to single-stranded RNAs. These studies revealed that a single GC dinucleotide in poly-uridine is sufficient for MBNL1 binding and that a second GC dinucleotide confers higher affinity MBNL1 binding. However additional GC dinucleotides do not enhance RNA binding. We also showed that the RNA sequences adjacent to the GC dinucleotides play an important role in MBNL1 binding with the following preference: uridines >cytidines >adenosines >guanosines. For high affinity binding by MBNL1, the distance between the two GC dinucleotides can vary from 1 to 17 nucleotides.

**Conclusions:**

These results suggest that MBNL1 is highly flexible and able to adopt different conformations to recognize RNAs with varying sequence configurations. Although MBNL1 contains four ZnFs, only two ZnF - GC dinucleotide interactions are necessary for high affinity binding.

## Background

Alternative pre-mRNA splicing significantly increases genome diversity with recent measurements suggesting that greater than 90% of genes undergo alternative splicing [1-3]. The different protein isoforms generated through alternative splicing can alter function or cellular localization, or it may provide a mechanism to regulate the levels of the protein by leading to non-productive splicing and RNA turnover (reviewed in [4,5]). Regulated alternative splicing is dependent upon the alternative splicing factors present in specific cell types or at different developmental stages. Many of these alternative splicing factors appear to regulate hundreds of exons and they function by recognizing specific RNA sequence elements within or near the regulated exons (reviewed in [6]).

Muscleblind-like 1 (MBNL1) is an alternative splicing factor that is associated with the disease myotonic dystrophy (DM). Patients with DM have a CTG or CCTG repeat expansion in an untranslated region of their genome. For DM type 1 (DM1), the CTG repeats are found in the 3' UTR of the DMPK gene, and for DM type 2 (DM2), the CCTG repeats are found within intron 1 of the CCHC-type zinc finger (ZnF), nucleic acid binding protein (CNBP) gene [7,8]. These CTG and CCTG repeats, when transcribed, form CUG/CCUG RNA stem loops that bind and sequester MBNL1 into nuclear foci resulting in a loss of normal MBNL1 function in the cell (reviewed in [9]). In studying the molecular basis of DM and the role MBNL1 plays, it was discovered that MBNL1 regulates the alternative splicing of a host of developmentally regulated transcripts [10-13]. In the cells of DM patients, adult splicing patterns revert back to embryonic splicing patterns and these incorrect splicing events are thought to cause many of the symptoms of the disease (reviewed in [9]).

Several MBNL1 binding sites have been identified, and a few of these have been characterized [10,14-17]. Complicating the analysis of MBNL1 binding to these sites and the CUG and CCUG repeats has been differentiating between the effects of RNA structure on MBNL1 binding and sequence specific RNA binding. MBNL1 interacts with RNA through its four CCCH ZnFs, although it is possible that other regions of the protein interact with RNA as well or play an important role in regulating splicing [18]. The ZnFs of MBNL1 fold into two compact domains ZnFs1/2 and ZnFs3/4 [19,20]. A crystal structure of ZnFs3/4 in complex with a 6 mer RNA (CGCUGU) revealed that these ZnFs bind single-stranded RNA through specific recognition of the Watson-Crick face of the GC dinucleotide [20]. Each ZnF (3 and 4) interacts with RNA in a similar manner and the structure of the ZnFs domain in complex with RNA lead Teplova and Patel to propose that MBNL1 binds RNA in a looped conformation [20]. This model predicts that for each ZnF to bind a GC dinucleotide a significant RNA linker must separate the GC dinucleotides because the RNA binding surfaces of ZnFs3/4 (or 1/2) are on opposing sides of the domain [20]. Binding to RNAs containing three different RNA linkers of 5, 10 and 15 nucleotides between the GC dinucleotides to ZnFs3/4 of MBNL1 supported this model [20]. However these experiments were only performed with the isolated ZnFs3/4 and it was unclear if the spacer would be important in the context of the four ZnFs of MBNL1. To determine if the spacing between GC dinucleotides was important for MBNL1 binding and to determine if the sequence of the RNA linker between the GC dinucleotides affected RNA binding, a series of single-stranded RNAs varying linker distances and different linker sequences were studied with MBNL1.

## Results and discussion

To better understand the RNA binding specificity of human MBNL1, we used a version of the protein that contains amino acids 2-260. Previous studies have shown that MBNL1 2-260, which contains all four ZnFs, binds RNA as well as the corresponding full-length protein [14]. This version of the protein was used due to its ease in expression and purification in a bacterial system. This region of the protein has been shown to be sufficient for splicing regulation [18].

### Determination of the number of GC dinucleotides required for high affinity binding by MBNL1

**Figure 1:**
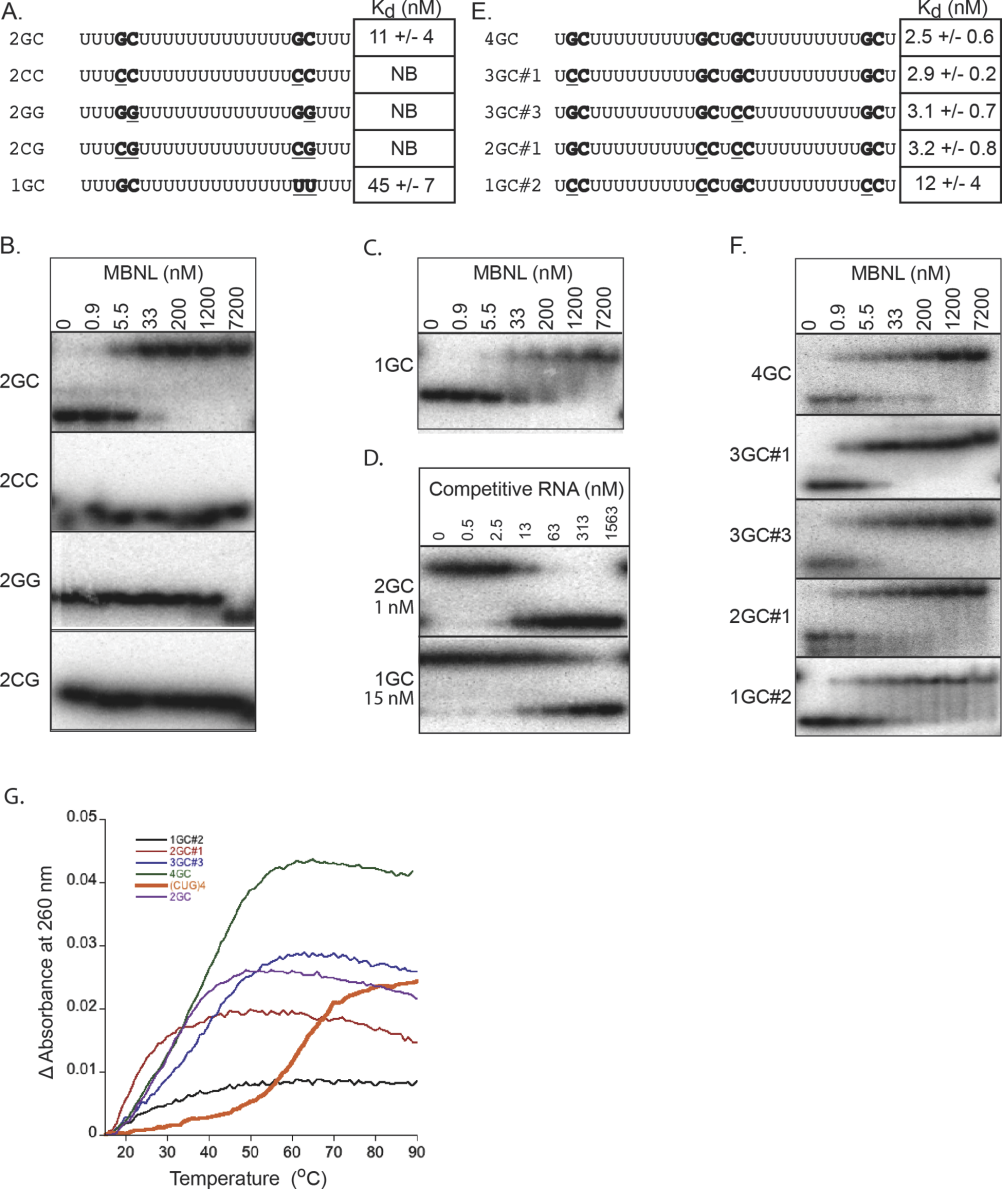
**The importance of GC dinucleotides in the context of single-strand RNA for MBNL1 binding**. A and E) Sequence of RNAs used in the gel shift assays to determine the specificity for the GC dinucleotides. GC dinucleotides are shown in bold with the subsequent mutations underlined. B, C and F) Representative gel shift assays. RNAs bound to MBNL1 (2-260) are labeled on the left. Concentrations of either MBNL1 or competitive RNA in each lane are at the top of the columns. D) Representative competition assay. Each lane contains 33.3 nM MBNL1, 0.02 nM radioactive 2GC RNA and increasing concentrations of competitive RNA. The identity of the competitive RNA is labeled on the left side of the gels and concentration of the competitive RNA (nM) is shown at the top of the column. On the left of each gel is the K_d _based upon a competition model of binding. G) Thermal denaturation analysis from representative RNAs monitored at 260 nm. The black line is 1GC#2, red is 2GC#1, blue is 3GC#3, green is 4GC, orange is (CUG)_4_, and purple is 2GC. The (CUG)_4 _sequence is GCUGCUGUUCGCUGCUG. Refer to part A and E for the sequences of the other RNAs.

According to the crystal structure of the MBNL1 ZnFs3/4 - RNA complex [20], the primary determinant of RNA specificity is the recognition of the Watson-Crick faces of the GC dinucleotide by the ZnFs. To test the importance of the GC dinucleotide in the context of single-stranded RNA for binding to MBNL1, one to four of these motifs were placed into a poly-uridine RNA (Figure 1A). Uridines were chosen as the template because we had previously shown that MBNL1 generally prefers uridines compared to the other three nucleotides [21]. Additionally, poly-uridine lacks RNA structure so the role of RNA structure in binding can be minimized allowing the focus to be on the role of specificity in a single-stranded RNA substrate.

To determine if these RNAs are single-stranded, a thermal denaturation assay was used to monitor the structure of these RNAs. For all RNAs tested with one to four GC dinucleotides, the melting point was below 40°C (Figure 1G), while the (CUG)_4 _RNA which was previously shown to be structured and bind MBNL1 [14] has a melting point of 60.6 +/- 0.6°C (Figure 1G). These thermal denaturation results indicate that these model RNAs are primarily single-stranded and can be used to test MBNL1's specificity in the absence of RNA structure. A possibility we cannot discount is that when MBNL1 binds RNA it could stabilize weak RNA structures; therefore we cannot completely rule out the role of RNA structure. Further experiments would be needed to explore this possibility.

As expected, when both GC dinucleotides are mutated to any other sequence or their polarity is reversed (2CC, 2GG and 2CG), MBNL1 binding is eliminated at the concentrations of protein tested (Figure 1B). Although we had found previously that MBNL1 prefers uridines over the other three nucleotides [21], these results showed that MBNL1 does not bind to long stretches of uridines alone with appreciable affinity.

Surprisingly, the presence of a single GC dinucleotide was sufficient for high affinity binding by MBNL1 (Figure 1C). The 1GC RNA bound with a K_d _of 45 nM while the 2GC RNA bound with a K_d _of 11 nM. These results indicate that MBNL1 requires only one GC dinucleotide for one of its four ZnFs to achieve significant RNA binding. Although the 1GC RNA bound with relatively high affinity, the complex appeared to dissociate during the gel shift assay based on the observed smearing (Figure 1C). To confirm the gel shift assay results, a competition assay was used. In this assay, MBNL1 was pre-bound to a radioactive 2GC RNA to form a stable complex. Then increasing amounts of cold competitive RNA were added (Figure 1D). The competition results showed a larger difference between the 1GC and 2GC RNAs compared to the binding assays (15-fold in the competition assay versus 4-fold in the gel shift assay); however these results are consistent with the gel shift assay and further demonstrate that MBNL1 only requires one GC dinucleotide for nanomolar affinity binding by MBNL1 to RNA. The differences in relative affinities between the 2GC and 1GC RNAs in the gel shift and competition assays may be due to the instability of the 1GC-MBNL complex making quantification of this complex in the gel shift assay difficult to measure accurately.

To determine if more than two GC dinucleotides increased RNA binding by MBNL1, a longer RNA (29 nucleotides, Figure 1E) containing up to four GC dinucleotides was used for binding studies. The oligonucleotides with two, three, or four dinucleotides all behaved in a similar manner (Figure 1F). MBNL1 formed a single complex with all RNAs and the K_d_s were all within error of each other (2.5 to 4.0 nM). A RNA containing one GC dinucleotide (1GC#2) was also tested in the context of the longer RNA and it showed an approximately 5-fold increase in K_d _with lower complex stability (smearing) as seen with the shorter 1GC RNA (Figure 1, compare 1GC and 1GC#2). These results indicate that adding more than two GC dinucleotides did not enhance MBNL1 binding to RNA in this context.

The observation that RNAs containing three or four GC dinucleotides didn't bind MBNL1 better than RNAs containing just two GC dinucleotides was surprising. It suggests that only two of the four ZnFs require GC motifs for high affinity binding while the other two ZnFs may bind other sequences such as a poly-uridine or are involved in an intramolecular interaction that precludes RNA binding. This intramolecular interaction is supported by the MBNL1 ZnFs3/4 crystal structure which formed a ZnF4 - ZnF4 interaction with a 1320 Å^2 ^interface that would preclude ZnF4 from binding RNA [20]. Thus we might predict that ZnF2 and ZnF4 are involved in a higher order structure while ZnF1 and ZnF3 are involved in RNA binding. Although it is possible that the two ZnFs involved in RNA binding could be any of the four ZnFs, as none of the ZnFs alone have been directly tested for RNA binding. In comparing this model with the known endogenous MBNL1 targets, it can be seen that many of these binding sites contain only two YGCY motifs suggesting that a mode in which MBNL1 uses only two of its ZnFs to bind YGCY motifs could be a common type of interaction for MBNL1 [10,14-16].

An alternative explanation for the lack of increased MBNL1 binding to more than two GC motifs is that the RNA we used may not allow all four GC dinucleotides to bind all four ZnFs. However, in RNA 3GC#3 the three GC dinucleotides are spaced by nine and twelve nucleotides, and the model proposed by Teplova and Patel describing MBNL1 binding to RNAs with multiple GC dinucleotides suggests that this type of spacing would allow for binding by three of the four ZnFs [20]. Our data described later also suggests that an RNA containing two GC dinucleotides with only a single nucleotide spacing does not result in a dramatic decrease in binding. This supports the idea that the spacing of the GC dinucleotides was not a problem in these RNA constructs.

### The role of nucleotides adjacent to the GC dinucleotides in MBNL1 RNA binding

**Figure 2:**
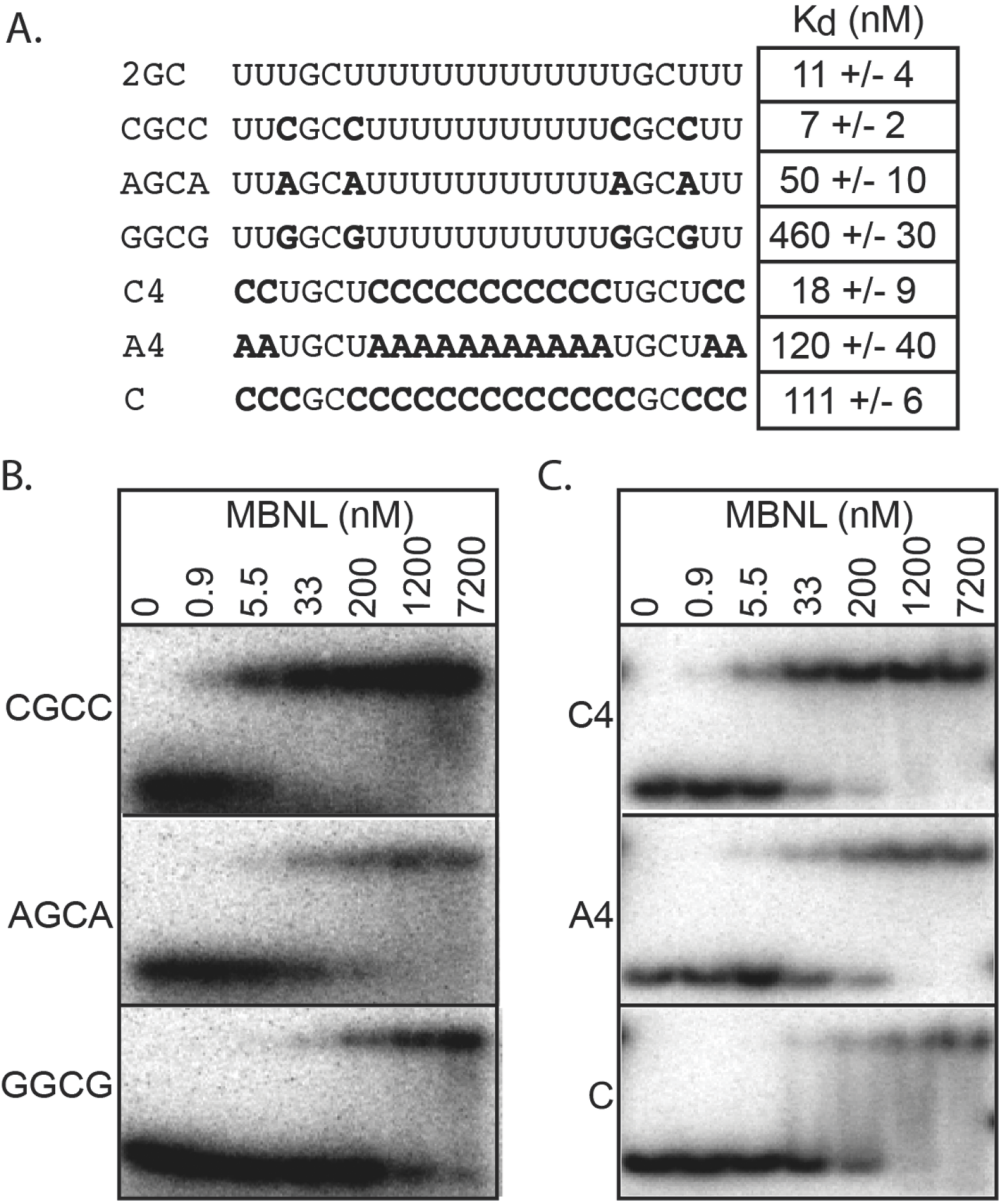
**Determination of the importance of nucleotides adjacent to the GC dinucleotides for MBNL1 binding**. A) RNAs used to analyze the sequence outside of the GC dinucleotides. Nucleotides that have been changed from the 2GC RNA are in bold. Next to the RNAs are the corresponding K_d_s, averaged over at least three independent experiments. B and C) Representative gel shifts assays. MBNL1 concentrations are at the top of each column. RNA being tested is on the left side of the gels.

Our previous research showed enrichment for YGCY in a SELEX assay of MBNL1 2-260, however the importance of the pyrimidines upstream and downstream of the GC was never directly tested [21]. Therefore we set about to determine the importance of nucleotides upstream and downstream of the GC dinucleotide by making mutations to these nucleotides in the context of the 2GC RNA (Figure 2A). The CGCC RNA bound as well as the 2GC RNA, with K_d_s of 7 nM and 11 nM respectively (Figure 2B). Replacing all of the uridines with cytidines decreased binding by 10-fold (Figure 2C, RNA C with a K_d _of 111 nM). However if the upstream and downstream cytidines were replaced with uridines (RNA C4, Figure 2C), wild type levels of RNA binding were restored. These results suggest that MBNL1 requires some uridines to be near the GC dinucleotides but that there is some flexibility in their location relative to the GC motifs.

Replacing the pyrimidines with purines between the GC motifs significantly reduced MBNL1 binding. The presence of adenosines adjacent to the GC dinucleotide weakened binding to MBNL1 by 5-fold while guanosines in these positions reduced binding by 40-fold (Figure 2B, RNAs AGCA and GGCG). Placing two UGCU motifs into a polyA RNA resulted in more than a 10-fold reduction in binding compared to the 2GC RNA (Figure 2C, A4 RNA), indicating that positions outside of the UGCU motifs also affect MBNL1's ability to bind RNA.

Our characterization of MBNL1 binding specificity is consistent with our previously proposed MBNL1 binding site motif of YGCY [21]. However, there seems to be an additional requirement of a uridine rich context as seen by the 10-fold decrease in binding between a cytidine rich and a uridine rich environment. This YGCY and U-rich requirement is not explained by the crystal structure of ZnFs3/4 in complex with RNA. In this structure ZnF3 only interacts with the GC dinucleotide and ZnF4 makes one base specific contact with the downstream uridine, therefore recognizing a GCU motif [20]. One explanation for this apparent discrepancy between our results and the crystal structure is that important protein-RNA contacts were not captured in the crystal structure. It is possible that ZnFs1/2 makes different RNA contacts than ZnFs3/4 or amino acids outside of the crystallized domains interact with RNA. Further studies are required to determine which regions of the protein are responsible for these additional RNA sequence requirements.

### The effects of the separation between GC dinucleotides in MBNL1 RNA binding

**Figure 3:**
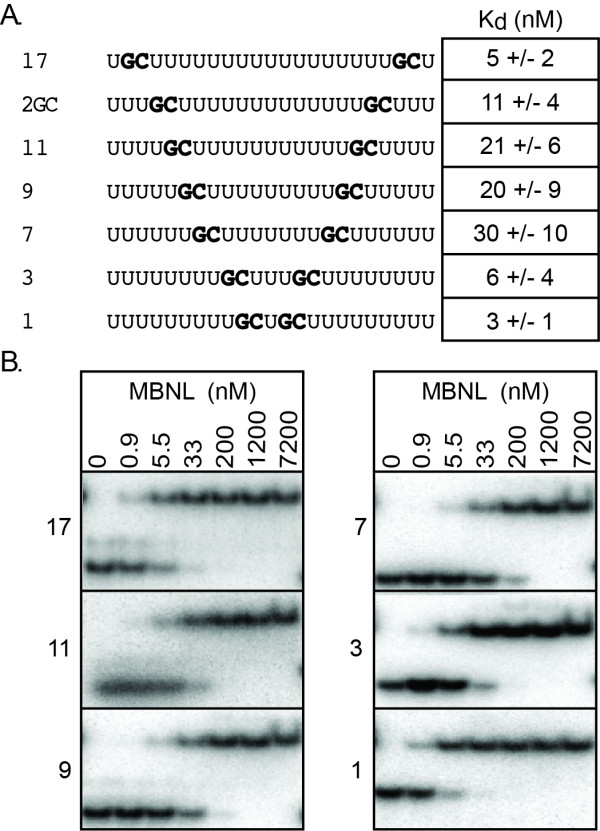
**Analysis of RNA spacing between GC dinucleotides on MBNL1 binding**. A) RNAs used in analysis of GC spacing. Name of RNA indicates the number of nucleotides between the two GC dinucleotides. GC dinucleotides are in bold. Next to each RNA is the corresponding K_d_, averaged over at least three independent experiments. B) Representative results from the gel shift assays. The RNA being tested is to the right of each gel and at the top of the column is the concentration of protein (nM) in each lane.

The crystal structures of the individual ZnFs domains of MBNL1 suggested that a RNA linker of 20 Å would be required to allow two ZnFs in one domain to each bind a GC dinucleotide [20]. In support of this model, Teplova and Patel showed that decreasing the GC dinucleotide linker from 15 or 10 nucleotides to 5 nucleotides reduced RNA binding with the ZnFs3/4 domain [20]. To determine if the distance between the two GC dinucleotides affects the binding of MBNL1 containing four ZnFs we systematically varied the distance between the GC motifs in the 2GC RNA (Figure 3A). Interestingly, the RNAs that bound MBNL1 with the highest affinity (K_d_s of 4-6 nM) had the longest and shortest linkers (Figure 3B). The RNAs with intermediate length RNA linkers of seven to 11 nucleotides bound MBNL1 with K_d_s between 20 and 30 nM. MBNL1's ability to bind all of these RNAs with relatively high affinity suggests that the two ZnFs domains can adopt different orientations to recognize the GC dinucleotides. The weaker binding to the RNAs containing intermediate length RNA linkers could be due to MBNL1 needing to adopt a slightly less favorable conformation to bind the RNA compared to the conformation adopted when binding short or long linker RNAs.

**Figure 4:**
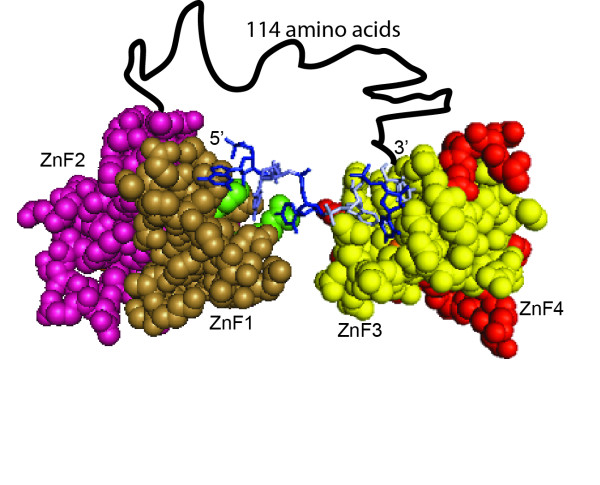
**Model of MBNL1 binding RNA**. Space-filling and stick model of MBNL1 ZnFs3/4 with CGCUGU RNA (PDB 3D2S) and MBNL1 ZnFs1/2 (PDB 3D2N). Tan = ZnF1, Magenta = ZnF2, Yellow = ZnF3, Red = ZnF4. Nucleotides making base specific contacts to ZnF3 are in white. These nucleotides come from two separate RNAs within the crystallized structure. Light blue is a cytidine from the ZnFs3/4 structure that has been modeled into the proposed cytidine binding pocket (green) of ZnF1, based upon sequence alignment with ZnFs3/4. The amino acid linker between ZnFs1/2 and ZnFs3/4 is modeled as a black line.

In MBNL1, the ZnFs domains are separated by 114 amino acids and we propose that this region of the protein provides flexibility that allows the ZnFs domains to bind RNAs with a wide range of spacing between the primary GC motifs. Figure 4 shows a model in which the ZnFs domains are oriented such that ZnF1 and ZnF3 are binding an RNA with a single nucleotide spacer between the GC dinucleotides. Although ZnFs3/4 was crystallized with a single GC RNA, there are multiple RNAs bound to this domain in the crystal structure. This structure allowed us to model how MBNL1 might bind an RNA containing two GC dinucleotides, assuming that the two RNAs observed in the crystal structure would normally be found in one longer RNA. The orientation of the ZnFs domains shown in Figure 4 may favor ZnFs domain-domain interactions that could contribute to the high affinity RNA binding seen with the 1 and 3 nucleotide spacer RNAs (Figure 3A). Other orientations of the ZnFs domains are possible such that ZnF2 and ZnF4 bind the RNA for example. The flexibility of MBNL1 may explain why it is able to recognize a diverse set of RNA targets that include the toxic CUG/CCUG repeats and many different sites within pre-mRNAs.

The proposed model explains how MBNL1 may recognize a wide range of single-stranded RNA targets, but it does not address how MBNL1 interacts with structured RNAs such as the CUG/CCUG repeats or stem-loop targets found in its pre-mRNA targets. It is possible that MBNL1 binds the YGCY motifs in the structured RNAs when these nucleotides are accessible (GC dinucleotides flipped out so the Watson-Crick face of the GC dinucleotides can be bound by the ZnFs domains) in these RNA targets. The flexibility of the linker between the ZnFs domains would allow MNBL1 to easily reach two sites on different parts of a structured RNA. Alternatively, MBNL1 may recognize its structured RNA targets using a different mode of recognition. In the future biochemical approaches using techniques in addition to the gel shift assay and structural studies will be necessary to determine if MBNL1 use one or more modes of binding to recognize single-stranded and structured RNA substrates.

## Conclusions

This study suggests that the ZnFs domains of MBNL1 are linked together by a highly flexible linker (Figure 4), which allows MBNL1 to recognize a wide variety of RNA targets. Although MBNL1 contains four ZnFs, it appears that only two ZnFs binding GC motifs are necessary for high affinity RNA binding.

## Materials and methods

### Protein Purification

The MBNL1 protein used in these studies contains amino acids 2-260 and was described previously [14]. Purification of the protein and removal of the GST tag was described previously [14] except that for our studies the protein was stored at -80°C rather than at -20°C.

### RNA synthesis, labeling, and purification

All RNAs were synthesized by Integrated DNA Technologies and 5' end labeled with [γ-^32^P]ATP. RNAs were then purified on a 10% denaturing polyacrylamide gel (19:1) and eluted into TE Buffer (10 mM Tris, 1 mM EDTA). RNA was then ethanol precipitated with glycogen and resuspended in TE Buffer. Finally a Bio-spin 6 column (Bio-Rad) equilibrated in TE Buffer was used.

### RNA thermal denaturation assay

1.5 μM RNA was incubated at 95°C for two minutes in 100 mM NaCl, 21 mM Tris 7.5, and 5 mM MgCl_2_. RNA was then incubated on ice for 30 minutes. The thermal denaturation analysis was performed on a Carey UV/VIS Spectrophotometer. The absorbance of the RNA was monitored at 260 nm while the temperature was increased from 15-90°C. Melting temperatures were determined by calculating the derivative of the absorbance versus temperature using the Cary program "Thermal". All RNAs were tested three times and T_m _values compared. The T_m _of all the RNAs used in these studies were measured at below 40°C while the (CUG)_4 _RNA was measured with a T_m _of 60.6 +/- 0.6°C.

### Gel mobility shift assay

RNA was initially heated to 95 °C for two minutes and then placed directly onto ice. RNA was then mixed with MBNL1 at concentrations indicated in the figures. The final reaction conditions were 100 mM NaCl, 21 mM Tris pH 7.5, 0.1 mM EDTA, 2 mg/mL BSA, 0.1 mg/mL Heparin, 0.5 mM BME, 5 mM DTT, 0.02% Triton X-100, 5 mM MgCl_2_, 10% glycerol, 0.02% bromophenol blue, and 0.02 nM RNA. The samples were incubated at room temperature for 30 minutes and then loaded onto a pre-chilled 6% polyacrylamide (19:1) gel running at 50 volts. Once the samples were loaded the voltage was increased to 150 volts and the gel was run for 30 minutes. After running, the gel was dried and exposed overnight.

Quantitation of the binding curve was described previously [14]. The data was fit using the equation  where m_1 _= a fitting factor, m_0 _= nM protein concentration, and m_2 _= nM K_d_.

### Competition assay

Competition assays were run under the same conditions as the gel shift assays except that the protein and radioactive RNA were incubated for 20 minutes and then the competitive RNA was added to the reaction and allowed to equilibrate for 30 minutes.

Quantitation of the competition assays was done using Kaleidagraph. The data was fit to the equation where m_2 _= competitive RNA K_d_, m_1 _= K_d _to radiolabeled RNA, P_t _= total protein added in the reaction, and m_0 _= amount of competitive RNA. When the competitive RNA and the radiolabeled RNA were the same, the equation was simplified to , assuming m_2 _and m_1 _are the same.

### Modeling of MBNL1 binding RNA

PDB files 3D2N and 3D2S were used to create Figure 4. Each PDB file was opened in separate Pymol windows. 3d2S contained ZnFs3/4 bound to RNA. The structure originally contained a ZnFs3/4 dimer bound to RNA. The extra ZnFs3/4 was removed and the RNA bound to ZnF4 was removed for the figure. In the other window the ZnFs1/2 structure (3D2N) was oriented to place the hypothesized cytidine binding pocket towards the unbound cytidine from 3D2S. Figures were then made of each of these windows and were merged in Photoshop. The amino acid linker was then added by linking the C-terminal amino acid from ZnFs1/2 with the N-terminal amino acid from ZnFs3/4.

## Authors' contributions

DC performed most of the experiments, data analysis, and drafted the manuscript. RH, PB, and KJ performed many of the replicate binding assays. DPG performed the thermal melt analysis. JAB conceived of the project, analyzed data and edited the manuscript. All authors read and approved the final manuscript.
